# Crystal structure of 1-[(2S*,4R*)-6-fluoro-2-methyl-1,2,3,4-tetra­hydro­quinolin-4-yl]pyrrolidin-2-one

**DOI:** 10.1107/S1600536814019254

**Published:** 2014-08-30

**Authors:** P. S. Pradeep, S. Naveen, M. N. Kumara, K. M. Mahadevan, N. K. Lokanath

**Affiliations:** a Department of Chemistry, Kuvempu University, Jnanasahyadri, Shankaraghatta 577 451, India; b Institution of Excellence, University of Mysore, Manasagangotri, Mysore 570 006, India; c Department of Chemistry, Yuvaraja’s College, University of Mysore, Mysore 570 005, India; d Department of Studies in Physics, University of Mysore, Manasagangotri, Mysore 570 006, India

**Keywords:** crystal structure, tetra­hydro­quinoline, pyrrolidine, chirality

## Abstract

In the title compound, C_14_H_17_FN_2_O, the 1,2,3,4-tetra­hydro­pyridine ring of the quinoline moiety adopts a half-chair conformation, while the pyrrolidine ring has an envelope conformation with the central methyl­ene C atom as the flap. The pyrrolidine ring lies in the equatorial plane and its mean plane is normal to the mean plane of the quinoline ring system, with a dihedral angle value of 88.37 (9)°. The bridging N—C bond distance [1.349 (3) Å] is substanti­ally shorter than the sum of the covalent radii (*d*_cov_: C—N = 1.47 Å and C=N = 1.27 Å), which indicates partial double-bond character for this bond, resulting in a certain degree of charge delocalization. In the crystal, mol­ecules are linked by N—H⋯O and C—H⋯O hydrogen bonds, forming sheets lying parallel to (10-1). These two-dimensional networks are linked *via* C—H⋯F hydrogen bonds and C—H⋯π inter­actions, forming a three-dimensional structure.

## Abstract 2

In the title compound, the 1,2,3,4-tetra­hydro­pyridine ring of the quinoline moiety adopts a half-chair conformation while the pyrrolidine ring has an envelope conformation. In the crystal, mol­ecules are linked by N—H⋯O and C—H⋯O hydrogen bonds, forming sheets lying parallel to (10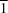), which are linked *via* C—H⋯F hydrogen bonds and C—H⋯π inter­actions, forming a three-dimensional structure.

## Chemical context   

Tetra­hydro­quinolines have been significant synthetic targets due to their ubiquitous distribution in natural products and as medicinal agents (Trost *et al.*, 1991 ▶). They are potential anti­cancer agents and 2-aryl-4-(2-oxopyrrolidin-1-yl)-1,2,3,4-tetra­hydro­quinolines have been reported to be inhibitors of HIV transcription. Furthermore, 2-methyl tetra­hydro­quino­lines have also been found to exhibit high modulating activity in multidrug resistance (MDR) (Hiessbock *et al.*, 1999 ▶). In view of their broad spectrum of medicinal properties and in continuation of our work on new quinoline-based therapeutic agents (Pradeep *et al.*, 2014 ▶), we have synthesized the title compound and report herein on its spectroscopic and crystallographic characterization.
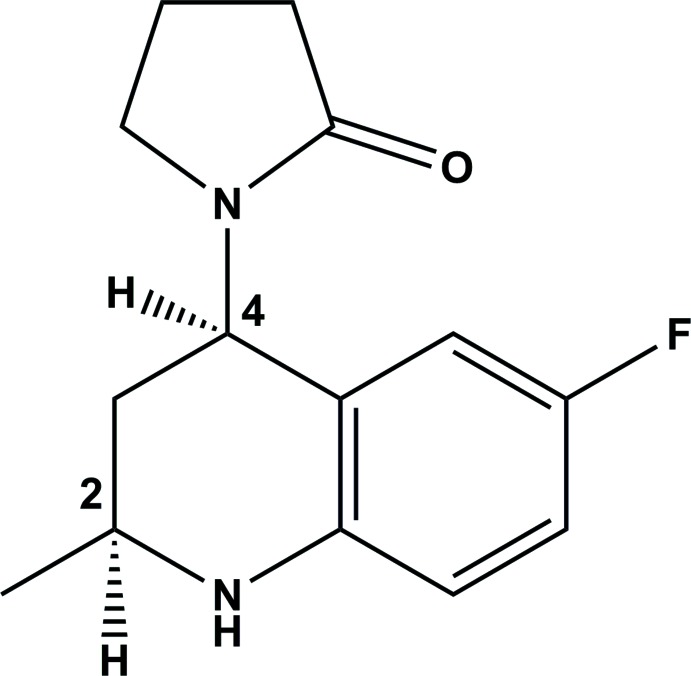


## Structural commentary   

**Figure 1:**
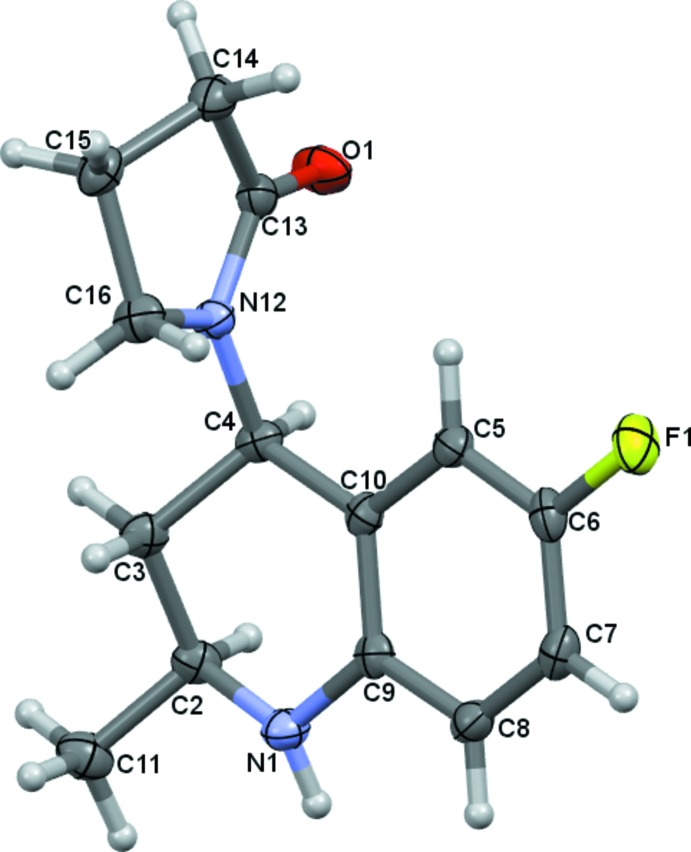
A view of the mol­ecular structure of the title mol­ecule, with the atom labelling. Displacement ellipsoids are drawn at the 50% probability level.

The mol­ecular structure of the title mol­ecule is shown in Fig. 1 ▶. The relative configuration of the asymmetric centers is *S* for atom C2 and *R* for atom C4.

The pyrrolidine ring adopts an envelope conformation with the flap atom C15 deviating by 0.197 (2) Å from the mean plane defined by the atoms N12/C13/C14/C16. The pyrrolidine ring lies in the equatorial plane and its mean plane is perpendicular to the mean plane of the quinoline ring system, as indicated by the dihedral angle of 88.37 (9)°. The N12—C13 distance [1.349 (3) Å] is substanti­ally shorter than the sum of the covalent radii [*d*_cov_: C—N = 1.47 Å and C=N = 1.27 Å; Holleman *et al.*, 2007 ▶], which indicates partial double-bond character for this bond, resulting in a certain degree of charge delocalization. The C13=O1 bond length of 1.235 (3) Å confirms the presence of a keto group in the pyrrolidine moiety.

The tetra­hydro­pyridine ring of the quinoline system adopts a half-chair conformation with atom C10 deviating by 0.285 (2) Å from the mean plane defined by atoms N1/C2–C4/C9. This is confirmed by the puckering amplitude *Q* = 0.496 (2) Å. Although the quinoline ring system adopts a distorted half-chair conformation, the torsion angles C9—N1—C2—C3 and C2—C3—C4—C10 are −40.8 (2) and −53.0 (2)°, respectively. These differ from the corresponding angles [−47.8 (2) and −45.0 (2)°, respectively] in 6-eth­oxy-1,2,3,4-tetra­hydro-2,2,4-tri­methyl­quinoline (Rybakov *et al.*, 2004 ▶). This can be attributed to the steric hindrance caused by the change in the substituents on the quinoline ring system.

The conformation of the tetra­hydro­pyridine ring and that of the pyrrolidine ring are similar to those observed in, for example, 1-[2-(2-fur­yl)-6-methyl-1,2,3,4-tetra­hydro­quinolin-4-yl]pyrrolidin-2-one (Vizcaya *et al.*, 2012 ▶).

## Supra­molecular features   

**Figure 2:**
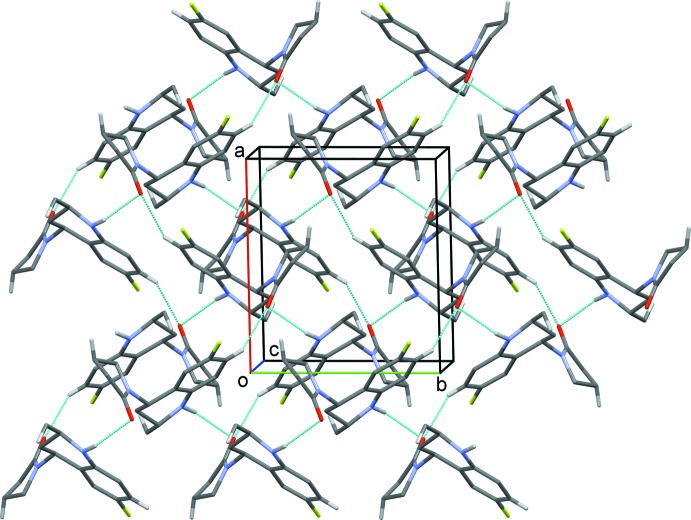
A viewed along the *c* axis of the crystal packing of the title compound. Hydrogen bonds are shown as dashed lines (see Table 1 ▶ for details; H atoms not involved in hydrogen bonding have been omitted for clarity).

**Figure 3:**
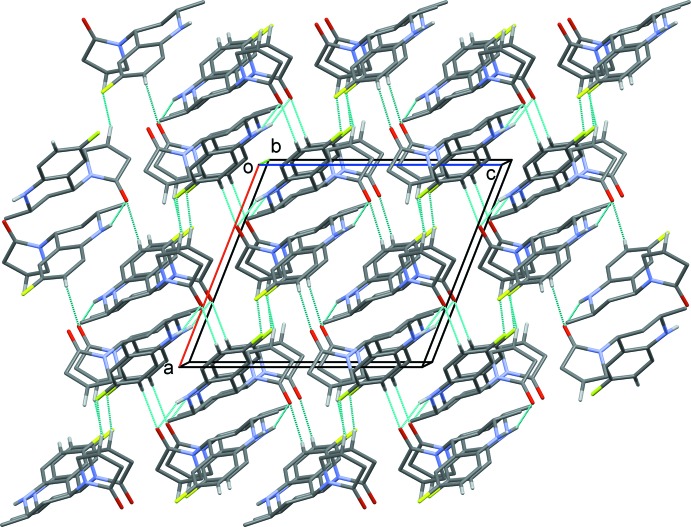
A viewed along the *b* axis of the crystal packing of the title compound. Hydrogen bonds are shown as dashed lines (see Table 1 ▶ for details; H atoms not involved in hydrogen bonding have been omitted for clarity).

In the crystal, mol­ecules are linked by N—H⋯O and C—H⋯O hydrogen bonds, forming sheets lying parallel to (10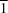); see Fig. 2 ▶ and Table 1 ▶. These two-dimensional networks are linked *via* C—H⋯F hydrogen bonds and C—H⋯π inter­actions, forming a three-dimensional structure (Table 1 ▶ and Fig. 3 ▶).

## Database survey   

A search of the Cambridge Structural Database (Version 5.35, last update May 2014; Allen *et al.*, 2002 ▶) for the substructure (1,2,3,4-tetra­hydro­quinolin-4-yl)pyrrolidin-2-one yielded seven hits. Two of these crystallized in a chiral space group; *P*2_1_2_1_2_1_ for the 2-(4-meth­oxy­phen­yl) derivative (refcode: HABXIT; Shen & Ji, 2008 ▶), and *P*6_1_ for the *trans* diastereomer of the 2-(4-nitro­phen­yl)-5-(5-phenyl-1,2-oxazol-3-yl) derivative (refcode: IKAZEA; Gutierrez *et al.*, 2011*a* ▶). The crystal structure of the racemic form of the latter has also been reported (refcode: QALCOX; Gutierrez *et al.*, 2011*b* ▶).

In all seven compounds, the tetra­hydro­pyridine ring has a half-chair conformation, while in three mol­ecules the pyrrolidine ring has an envelope conformation and in another three mol­ecules a twist conformation. The orientation of the pyrrolidine ring with respect to the quinoline ring is very similar if one excludes the two compounds that have a substituent in the 5-position of the quinoline ring (Gutierrez *et al.*, 2011*a* ▶,*b* ▶). The two mean planes are inclined to one another by dihedral angles varying from *ca* 79.98 to 89.59°, compared to 88.37 (9)° in the title compound.

## Synthesis and crystallization   

A catalytic amount of SbF_3_ (10 mol%) was added to a mixture of 4-flouroaniline (1 equivalent) and *N*-vinyl­pyrrolidone (2–3 equivalents) in aceto­nitrile (5–10 ml). The reaction mixture was stirred at ambient temperature (292 K) for 20–70 min. After completion of the reaction, as indicated by TLC using ethyl acetate/hexane as eluent, the solvent was removed under *vacuo*. The crude product was then quenched with water and the catalyst was decomposed by addition of the appropriate amount of sodium bicarbonate solution. It was then extracted with ethyl acetate (10 ml × 5 times), dried and purified by column chromatography using ethyl acetate/hexane as eluent (petroleum ether/ethyl acetate 80:20 *v*/*v*). White crystals were obtained by slow evaporation of the solvent.

In the ^1^H NMR spectrum of the title compound, the three quadrates at δ 1.60, 2.95 and 3.22 p.p.m. correspond to three protons at C_3_—H, C_5′_—H and C_4′_—H, respectively. A doublet at δ 5.24 p.p.m. corresponds to C_4_—H, a singlet at δ 5.62 p.p.m. corresponds to the –NH proton and the number of protons is in accordance with the obtained structure. Additional support to elucidate the structure was obtained from ^13^C NMR (see the archived CIF for more details). The mass spectrum was recorded as additional evidence for the proposed structure: *M*+1 peak at *m*/*z* = 250.1.

## Refinement   

Crystal data, data collection and structure refinement details are summarized in Table 2 ▶. The NH H atom was located from a difference Fourier map and freely refined. The C-bound H atoms were fixed geometrically (C—H = 0.93–0.96 Å) and allowed to ride on their parent atoms with *U*_iso_(H) = 1.5*U*_eq_(C) for methyl H atoms and = 1.2*U*_eq_(C) for other H atoms.

## Supplementary Material

Crystal structure: contains datablock(s) I. DOI: 10.1107/S1600536814019254/su2767sup1.cif

Structure factors: contains datablock(s) I. DOI: 10.1107/S1600536814019254/su2767Isup2.hkl

Supporting information file. DOI: 10.1107/S1600536814019254/su2767Isup3.cml

CCDC reference: 1021159

Additional supporting information:  crystallographic information; 3D view; checkCIF report

## Figures and Tables

**Table 1:** Hydrogen-bond geometry (Å, °) *Cg*1 is the centroid of the C5–C10 ring.

*D*—H⋯*A*	*D*—H	H⋯*A*	*D*⋯*A*	*D*—H⋯*A*
N1—H1*N*⋯O1^i^	0.84 (3)	2.46 (3)	3.273 (2)	162 (2)
C7—H7⋯O1^ii^	0.93	2.51	3.351 (3)	150
C15—H15*B*⋯F1^iii^	0.97	2.48	3.189 (3)	130
C11—H11*C*⋯*Cg*1^iv^	0.97	2.80	3.748 (3)	168

Symmetry codes: (i) 
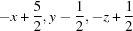
; (ii) 
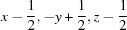
; (iii) 
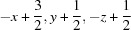
; (iv) 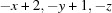.

**Table 2:** Experimental details

Crystal data	
Chemical formula	C_14_H_17_FN_2_O
*M*_r_	248.30
Crystal system, space group	Monoclinic, *P*2_1_/*n*
Temperature (K)	100
*a*, *b*, *c* (Å)	11.3414 (3), 9.1909 (3), 12.6799 (4)
β (°)	111.569 (2)
*V* (Å^3^)	1229.17 (7)
*Z*	4
Radiation type	Cu *K*α
μ (mm^−1^)	0.79
Crystal size (mm)	0.23 × 0.22 × 0.21

Data collection	
Diffractometer	Bruker X8 Proteum
Absorption correction	Multi-scan (*SADABS*; Bruker, 2013 ▶)
*T*_min_, *T*_max_	0.834, 0.848
No. of measured, independent and observed [*I* > 2σ(*I*)] reflections	8574, 2009, 1488
*R*_int_	0.071
(sin θ/λ)_max_ (Å^−1^)	0.585

Refinement	
*R*[*F* ^2^ > 2σ(*F* ^2^)], *wR*(*F* ^2^), *S*	0.043, 0.122, 1.00
No. of reflections	2009
No. of parameters	168
H-atom treatment	H atoms treated by a mixture of independent and constrained refinement
Δρ_max_, Δρ_min_ (e Å^−3^)	0.20, −0.22

Computer programs: *APEX2* and *SAINT* (Bruker, 2013 ▶), *SHELXS97* and *SHELXL97* (Sheldrick, 2008 ▶), *PLATON* (Spek, 2009 ▶), *Mercury* (Macrae *et al.*, 2008 ▶) and *publCIF* (Westrip, 2010 ▶)’.

## supplementary crystallographic information

### Crystal data

**Table:**

C_14_H_17_FN_2_O	*F*(000) = 528
*M**_r_* = 248.30	*D*_x_ = 1.342 Mg m^−^^3^
Monoclinic, *P*2_1_/*n*	Cu *K*α radiation, λ = 1.54178 Å
Hall symbol: -P 2yn	Cell parameters from 2009 reflections
*a* = 11.3414 (3) Å	θ = 4.5–64.4°
*b* = 9.1909 (3) Å	µ = 0.79 mm^−^^1^
*c* = 12.6799 (4) Å	*T* = 100 K
β = 111.569 (2)°	Block, white
*V* = 1229.17 (7) Å^3^	0.23 × 0.22 × 0.21 mm
*Z* = 4	

### Data collection

**Table:**

Bruker X8 Proteum diffractometer	2009 independent reflections
Radiation source: Bruker MicroStar microfocus rotating anode	1488 reflections with *I* > 2σ(*I*)
Helios multilayer optics monochromator	*R*_int_ = 0.071
Detector resolution: 18.4 pixels mm^-1^	θ_max_ = 64.4°, θ_min_ = 4.5°
φ and ω scans	*h* = −13→13
Absorption correction: multi-scan (*SADABS*; Bruker, 2013)	*k* = −10→10
*T*_min_ = 0.834, *T*_max_ = 0.848	*l* = −14→14
8574 measured reflections	

### Refinement

**Table:**

Refinement on *F*^2^	Primary atom site location: structure-invariant direct methods
Least-squares matrix: full	Secondary atom site location: difference Fourier map
*R*[*F*^2^ > 2σ(*F*^2^)] = 0.043	Hydrogen site location: inferred from neighbouring sites
*wR*(*F*^2^) = 0.122	H atoms treated by a mixture of independent and constrained refinement
*S* = 1.00	*w* = 1/[σ^2^(*F*_o_^2^) + (0.0682*P*)^2^] where *P* = (*F*_o_^2^ + 2*F*_c_^2^)/3
2009 reflections	(Δ/σ)_max_ = 0.049
168 parameters	Δρ_max_ = 0.20 e Å^−^^3^
0 restraints	Δρ_min_ = −0.22 e Å^−^^3^

### Special details

**Table:**

Experimental. ^1^H NMR was recorded at 400 MHz in Dimethylsulfoxide (DMSO-d_6_). ^13^C NMR was recorded at 400 MHz in DMSO-d_6_. Mass spectra was recorded on a Jeol SX 102=DA-6000 (10 kV) fast atom bombardment (FAB) mass spectrometer. ^1^H NMR(400 MHz, DMSO-d_6_): δ = 1.12 (s, 3H), 1.60 (q, J = 12.00 Hz, 1H), 1.72–1.74 (m, 1H), 1.89–1.91 (m, 2H), 2.26–2.28 (m, 2H), 2.95 (q, J = 6.80 Hz, 1H), 3.22 (q, J = 7.20 Hz, 1H), 3.41–3.43 (m, 1H), 5.24 (d, J = 5.60 Hz, 1H), 5.62 (s, 1H), 6.40–6.41 (m, 1H), 6.49–6.50 (m, 1H), 6.74–6.75 (m, 1H) p.p.m..^13^C NMR (400 MHz, DMSO-d_6_): δ = 17.6, 21.6, 30.6, 33.2, 41.6, 46.1, 47.2, 11.7, 114.4, 119.2, 142.9, 153.1, 155.4, 174.6 p.p.m..MS (70 eV) m/*z* (%): 250.1 (*M*^+^, 99.63)HPLC Purity = 97.9%.
Geometry. Bond distances, angles *etc*. have been calculated using the rounded fractional coordinates. All su's are estimated from the variances of the (full) variance-covariance matrix. The cell e.s.d.'s are taken into account in the estimation of distances, angles and torsion angles
Refinement. Refinement on *F*^2^ for ALL reflections except those flagged by the user for potential systematic errors. Weighted *R*-factors *wR* and all goodnesses of fit *S* are based on *F*^2^, conventional *R*-factors *R* are based on *F*, with *F* set to zero for negative *F*^2^. The observed criterion of *F*^2^ > σ(*F*^2^) is used only for calculating -*R*-factor-obs *etc*. and is not relevant to the choice of reflections for refinement. *R*-factors based on *F*^2^ are statistically about twice as large as those based on *F*, and *R*-factors based on ALL data will be even larger.

### Fractional atomic coordinates and isotropic or equivalent isotropic displacement parameters (Å^2^)

**Table:**

	*x*	*y*	*z*	*U*_iso_*/*U*_eq_	
F1	0.84526 (11)	0.14151 (14)	0.28243 (11)	0.0311 (4)	
O1	1.19100 (13)	0.59593 (18)	0.49292 (13)	0.0316 (5)	
N1	1.15416 (15)	0.3610 (2)	0.07073 (15)	0.0220 (6)	
N12	1.06638 (14)	0.62374 (18)	0.30482 (14)	0.0181 (5)	
C2	1.23865 (17)	0.4842 (2)	0.11812 (18)	0.0208 (6)	
C3	1.16936 (17)	0.5931 (2)	0.16463 (18)	0.0217 (6)	
C4	1.13482 (16)	0.5243 (2)	0.25882 (17)	0.0191 (6)	
C5	0.98300 (17)	0.3254 (2)	0.26777 (18)	0.0201 (7)	
C6	0.92150 (17)	0.1966 (2)	0.22961 (19)	0.0226 (7)	
C7	0.93354 (18)	0.1203 (2)	0.14035 (19)	0.0236 (7)	
C8	1.01288 (18)	0.1756 (2)	0.09018 (19)	0.0224 (7)	
C9	1.07908 (16)	0.3066 (2)	0.12729 (17)	0.0188 (6)	
C10	1.06312 (16)	0.3830 (2)	0.21747 (17)	0.0177 (6)	
C11	1.27895 (19)	0.5500 (3)	0.02753 (19)	0.0280 (7)	
C13	1.09465 (18)	0.6419 (2)	0.41705 (18)	0.0217 (7)	
C14	0.98829 (18)	0.7274 (2)	0.4329 (2)	0.0252 (7)	
C15	0.92345 (18)	0.7996 (2)	0.31843 (19)	0.0246 (7)	
C16	0.94501 (18)	0.6917 (2)	0.23558 (19)	0.0242 (7)	
H1N	1.182 (2)	0.296 (3)	0.039 (2)	0.033 (7)*	
H2	1.31410	0.44940	0.18040	0.0250*	
H3A	1.09290	0.62550	0.10410	0.0260*	
H3B	1.22290	0.67730	0.19400	0.0260*	
H4	1.21440	0.49960	0.32060	0.0230*	
H5	0.97130	0.37440	0.32730	0.0240*	
H7	0.88930	0.03410	0.11480	0.0280*	
H8	1.02290	0.12520	0.03040	0.0270*	
H11A	1.31770	0.47640	−0.00270	0.0420*	
H11B	1.33870	0.62680	0.05990	0.0420*	
H11C	1.20600	0.58850	−0.03210	0.0420*	
H14A	1.02120	0.79930	0.49250	0.0300*	
H14B	0.93040	0.66350	0.45110	0.0300*	
H15A	0.96160	0.89310	0.31530	0.0300*	
H15B	0.83370	0.81320	0.30230	0.0300*	
H16A	0.87740	0.62030	0.21000	0.0290*	
H16B	0.95120	0.74120	0.17030	0.0290*	

### Atomic displacement parameters (Å^2^)

**Table:**

	*U*^11^	*U*^22^	*U*^33^	*U*^12^	*U*^13^	*U*^23^
F1	0.0307 (6)	0.0272 (8)	0.0426 (9)	−0.0022 (5)	0.0220 (6)	0.0062 (6)
O1	0.0320 (8)	0.0396 (11)	0.0193 (9)	0.0119 (7)	0.0048 (7)	0.0021 (7)
N1	0.0231 (8)	0.0223 (11)	0.0230 (11)	−0.0002 (8)	0.0113 (8)	−0.0032 (9)
N12	0.0185 (8)	0.0178 (10)	0.0172 (10)	0.0019 (7)	0.0055 (7)	−0.0011 (8)
C2	0.0169 (9)	0.0252 (12)	0.0189 (12)	−0.0030 (8)	0.0048 (8)	−0.0023 (9)
C3	0.0197 (9)	0.0230 (12)	0.0216 (12)	−0.0038 (8)	0.0066 (9)	−0.0035 (9)
C4	0.0159 (9)	0.0208 (12)	0.0187 (12)	0.0019 (8)	0.0042 (8)	−0.0025 (9)
C5	0.0223 (10)	0.0180 (12)	0.0210 (12)	0.0035 (8)	0.0090 (9)	0.0017 (9)
C6	0.0206 (9)	0.0211 (12)	0.0279 (13)	0.0013 (9)	0.0112 (9)	0.0085 (10)
C7	0.0225 (10)	0.0161 (12)	0.0281 (13)	−0.0008 (8)	0.0046 (9)	0.0019 (10)
C8	0.0254 (10)	0.0178 (12)	0.0226 (12)	0.0018 (9)	0.0073 (9)	−0.0021 (9)
C9	0.0159 (9)	0.0185 (12)	0.0194 (12)	0.0051 (8)	0.0035 (8)	0.0040 (9)
C10	0.0157 (9)	0.0163 (12)	0.0190 (11)	0.0034 (8)	0.0040 (8)	0.0020 (9)
C11	0.0244 (10)	0.0359 (14)	0.0254 (13)	−0.0044 (10)	0.0112 (9)	−0.0019 (11)
C13	0.0256 (10)	0.0194 (12)	0.0213 (12)	−0.0030 (9)	0.0099 (9)	0.0008 (10)
C14	0.0281 (10)	0.0227 (13)	0.0290 (13)	−0.0002 (9)	0.0156 (9)	−0.0012 (10)
C15	0.0221 (10)	0.0215 (12)	0.0298 (13)	0.0024 (9)	0.0090 (9)	−0.0007 (10)
C16	0.0194 (9)	0.0266 (13)	0.0232 (12)	0.0066 (9)	0.0038 (9)	0.0000 (10)

### Geometric parameters (Å, º)

**Table:**

F1—C6	1.371 (2)	C14—C15	1.518 (3)
O1—C13	1.235 (3)	C15—C16	1.528 (3)
N1—C2	1.462 (3)	C2—H2	0.9800
N1—C9	1.392 (3)	C3—H3A	0.9700
N12—C4	1.453 (3)	C3—H3B	0.9700
N12—C13	1.349 (3)	C4—H4	0.9800
N12—C16	1.472 (3)	C5—H5	0.9300
N1—H1N	0.84 (3)	C7—H7	0.9300
C2—C11	1.510 (3)	C8—H8	0.9300
C2—C3	1.519 (3)	C11—H11A	0.9600
C3—C4	1.524 (3)	C11—H11B	0.9600
C4—C10	1.520 (3)	C11—H11C	0.9600
C5—C6	1.369 (3)	C14—H14A	0.9700
C5—C10	1.392 (3)	C14—H14B	0.9700
C6—C7	1.380 (3)	C15—H15A	0.9700
C7—C8	1.376 (3)	C15—H15B	0.9700
C8—C9	1.405 (3)	C16—H16A	0.9700
C9—C10	1.409 (3)	C16—H16B	0.9700
C13—C14	1.514 (3)		
			
C2—N1—C9	119.93 (17)	C2—C3—H3B	110.00
C4—N12—C13	123.12 (17)	C4—C3—H3A	110.00
C4—N12—C16	123.22 (16)	C4—C3—H3B	110.00
C13—N12—C16	112.59 (17)	H3A—C3—H3B	108.00
C2—N1—H1N	116.2 (17)	N12—C4—H4	107.00
C9—N1—H1N	113.5 (18)	C3—C4—H4	107.00
C3—C2—C11	112.08 (17)	C10—C4—H4	107.00
N1—C2—C3	108.38 (17)	C6—C5—H5	120.00
N1—C2—C11	109.48 (18)	C10—C5—H5	120.00
C2—C3—C4	110.45 (15)	C6—C7—H7	121.00
N12—C4—C10	112.27 (16)	C8—C7—H7	121.00
C3—C4—C10	110.07 (16)	C7—C8—H8	119.00
N12—C4—C3	112.50 (15)	C9—C8—H8	119.00
C6—C5—C10	120.03 (19)	C2—C11—H11A	109.00
F1—C6—C7	118.91 (17)	C2—C11—H11B	109.00
F1—C6—C5	118.54 (18)	C2—C11—H11C	109.00
C5—C6—C7	122.6 (2)	H11A—C11—H11B	110.00
C6—C7—C8	118.04 (18)	H11A—C11—H11C	109.00
C7—C8—C9	121.42 (19)	H11B—C11—H11C	110.00
N1—C9—C10	121.63 (17)	C13—C14—H14A	111.00
N1—C9—C8	119.20 (18)	C13—C14—H14B	111.00
C8—C9—C10	119.11 (18)	C15—C14—H14A	111.00
C4—C10—C9	119.59 (17)	C15—C14—H14B	111.00
C4—C10—C5	121.56 (17)	H14A—C14—H14B	109.00
C5—C10—C9	118.84 (17)	C14—C15—H15A	111.00
O1—C13—C14	126.5 (2)	C14—C15—H15B	111.00
N12—C13—C14	108.20 (18)	C16—C15—H15A	111.00
O1—C13—N12	125.3 (2)	C16—C15—H15B	111.00
C13—C14—C15	103.28 (18)	H15A—C15—H15B	109.00
C14—C15—C16	103.30 (16)	N12—C16—H16A	111.00
N12—C16—C15	102.49 (17)	N12—C16—H16B	111.00
N1—C2—H2	109.00	C15—C16—H16A	111.00
C3—C2—H2	109.00	C15—C16—H16B	111.00
C11—C2—H2	109.00	H16A—C16—H16B	109.00
C2—C3—H3A	110.00		
			
C9—N1—C2—C3	−40.8 (2)	C3—C4—C10—C5	−156.85 (18)
C9—N1—C2—C11	−163.35 (18)	C3—C4—C10—C9	24.2 (2)
C2—N1—C9—C8	−170.60 (18)	C10—C5—C6—F1	178.75 (18)
C2—N1—C9—C10	12.4 (3)	C10—C5—C6—C7	−1.1 (3)
C13—N12—C4—C3	−133.86 (19)	C6—C5—C10—C4	−178.91 (19)
C13—N12—C4—C10	101.3 (2)	C6—C5—C10—C9	0.1 (3)
C16—N12—C4—C3	58.9 (2)	F1—C6—C7—C8	−178.47 (19)
C16—N12—C4—C10	−66.0 (2)	C5—C6—C7—C8	1.4 (3)
C4—N12—C13—O1	11.8 (3)	C6—C7—C8—C9	−0.7 (3)
C4—N12—C13—C14	−168.15 (17)	C7—C8—C9—N1	−177.40 (19)
C16—N12—C13—O1	−179.74 (19)	C7—C8—C9—C10	−0.3 (3)
C16—N12—C13—C14	0.4 (2)	N1—C9—C10—C4	−3.4 (3)
C4—N12—C16—C15	−172.51 (17)	N1—C9—C10—C5	177.61 (19)
C13—N12—C16—C15	19.0 (2)	C8—C9—C10—C4	179.63 (18)
N1—C2—C3—C4	61.2 (2)	C8—C9—C10—C5	0.6 (3)
C11—C2—C3—C4	−177.83 (17)	O1—C13—C14—C15	160.3 (2)
C2—C3—C4—N12	−179.05 (16)	N12—C13—C14—C15	−19.8 (2)
C2—C3—C4—C10	−53.0 (2)	C13—C14—C15—C16	30.3 (2)
N12—C4—C10—C5	−30.7 (3)	C14—C15—C16—N12	−29.9 (2)
N12—C4—C10—C9	150.31 (18)		

### Hydrogen-bond geometry (Å, º)

Cg1 is the centroid of the C5–C10 ring.

**Table:**

*D*—H···*A*	*D*—H	H···*A*	*D*···*A*	*D*—H···*A*
N1—H1*N*···O1^i^	0.84 (3)	2.46 (3)	3.273 (2)	162 (2)
C7—H7···O1^ii^	0.93	2.51	3.351 (3)	150
C15—H15*B*···F1^iii^	0.97	2.48	3.189 (3)	130
C11—H11*C*···*Cg*1^iv^	0.97	2.80	3.748 (3)	168

Symmetry codes: (i) −*x*+5/2, *y*−1/2, −*z*+1/2; (ii) *x*−1/2, −*y*+1/2, *z*−1/2; (iii) −*x*+3/2, *y*+1/2, −*z*+1/2; (iv) −*x*+2, −*y*+1, −*z*.
